# Serum inflammatory cytokines levels and the correlation analyses in Parkinson’s disease

**DOI:** 10.3389/fcell.2023.1104393

**Published:** 2023-02-16

**Authors:** Jiajia Fu, Sihui Chen, Jiao Liu, Jing Yang, Ruwei Ou, Lingyu Zhang, Xueping Chen, Huifang Shang

**Affiliations:** Department of Neurology, West China Hospital, Sichuan University, Chengdu, Sichuan, China

**Keywords:** Parkinson’s disease, serum inflammatory cytokines, association analysis, IL-6, IL-8, TNF-α

## Abstract

**Objective:** To investigate the serum levels of inflammatory cytokines and the correlations with Parkinson’s disease (PD) clinical symptoms.

**Methods:** Serum levels of the cytokines, including IL-6, IL-8, and TNF-α, were measured in 273 PD patients and 91 healthy controls (HCs). The clinical manifestations of PD were assessed with nine different scales to evaluate the cognitive function, non-motor symptoms, motor symptoms, and disease severity. The differences in these inflammatory indicators were examined between PD patients and HCs, and the correlations of these inflammatory indicators with clinical variables were analyzed in PD patients.

**Results:** Serum levels of interleukin-6 (IL-6) and tumor necrosis factor-α (TNF-α) in PD patients were higher than those in HCs, but serum interleukin-8 (IL-8) level was not significantly different from that in HCs. In PD patients, serum IL-6 level was positively correlated with age of onset, the Hamilton Depression Scale (HAMD), and the Non-Motor Symptom Scale (NMSS), UPDRS part I, part II, and part III, but it was inversely correlated with the Frontal Assessment Battery (FAB) and the Montreal Cognitive Assessment (MoCA) scores. Serum TNF-α level was positively correlated with age of onset and H&Y stage in PD patients (*p* = .037), but negatively correlated with FAB scores in PD patients (*p* = .010). However, no associations were found between all the clinical variables and the serum IL-8 level. The forward binary logistic regression model revealed that serum IL-6 level was associated with MoCA (*p* = .023) and UPDRS I scores (*p* = .023), but no associations was found with the remaining factors. The ROC curve of TNF-α for the diagnosis of PD showed the area under the curve (AUC) was .719 (*p* < .05, 95% CI: .655–.784), and the critical value of TNF-α was 5.380 pg/ml, with a diagnostic sensitivity of 76.0% and a specificity of 59.3%.

**Conclusion:** Our results suggest increased serum levels of IL-6 and TNF-α in PD, we further found that IL-6 level was associated with non-motor symptoms and cognitive dysfunction, and IL-6 may play a role in the pathophysiology of non-motor symptoms in PD. At the same time, we also propose that TNF-α has a good diagnostic value for PD despite its irrelevance to clinical symptoms.

## Introduction

Parkinson’s disease (PD) is the second most common neurodegenerative disease. The main manifestations are motor symptoms, such as bradykinesia, myotonia, static tremor and postural balance disorder, accompanied by hyposmia, anxiety and depression, fatigue, sleep disturbance and other non-motor symptoms (Kalia and Lang, 2015). The pathogenesis of PD is not clear, but a large number of studies have shown that inflammatory response plays an important role in the pathological characteristics of PD. Glial cell activation and cytokines expression can promote the occurrence and development of PD. Previous studies measured the profile of serum or plasma inflammatory markers in PD patients. Some studies found that the levels of inflammatory factors in serum, cerebrospinal fluid (CSF), and brain tissue of PD were higher than those of healthy controls (HCs) (Ishizawa et al., 2008; Fernández-Botrán et al., 2011; Mosley et al., 2012; Wang et al., 2015; Tan et al., 2020). Still, some studies did not detect an elevation of serum inflammatory factor levels in PD patients (Stypuła et al., 1996; Koziorowski et al., 2012; Dursun et al., 2015; Williams-Gray et al., 2016). Tumor necrosis factor (TNFs), interleukin (IL) and other inflammatory factors are immune-activating key signal molecules that function in the brain and peripheral nerves (Tabas and Glass, 2013).

Numerous studies investigated the relationship between PD clinical symptoms and serum inflammatory factor levels, but the results were inconsistent, and both positive (Reale et al., 2009; Williams-Gray et al., 2016; Kouchaki et al., 2018; Rocha et al., 2018) and negative (Dufek et al., 2009; Koziorowski et al., 2012; Menza et al., 2010; Rocha et al., 2014; Herlofson et al., 2018) results have been reported. Therefore, the association between PD and serum cytokines remains controversial, and there is little research on the specific diagnostic value of related cytokines. The associations of serum inflammation factors with non-motor symptoms, especially cognitive impairment of PD were less explored since most of the studies have only focused on neuropsychiatric symptoms (Selikhova et al., 2002; Dufek et al., 2009; Menza et al., 2010; Rocha et al., 2014; Williams-Gray et al., 2016; Herlofson et al., 2018; Rocha et al., 2018; Lindqvist et al., 2012). A previous study reported that serum inflammatory factors may not be related to the cognitive function of PD, but this study only included 45 patients with PD (Kim et al., 2022).

The current study was designed: 1) to measure the levels of serum interleukin (IL)-6, IL-8, tumor necrosis factor-α (TNF-α) in PD patients and HCs and 2) to test whether these inflammatory indicators would correlate with disease characteristics.

## Patients and methods

A total of 273 PD patients were admitted to the departments of Neurology West China Hospital of Sichuan University from October 2019 to January 2022, and 91 HCs were also recruited. This study has been approved by the Ethical Committee of West China Hospital of Sichuan University and obtained the informed consent of the study subjects. PD patients met the diagnostic criteria of MDS (Postuma et al., 2015; Höglinger et al., 2017). The age and gender of HCs were matched with that of the PD group. Exclusion criteria o were listed as follows: 1) secondary parkinsonism syndrome caused by diseases such as brain trauma and cerebral infarction or taking drugs such as antipsychotics; 2) other extrapyramidal diseases such as MSA, PSP, CBD; 3) patients with acute or chronic inflammatory system or immune system diseases, co-existing various potential infections such as pulmonary infection, urinary tract infection, serious respiratory, circulatory, digestive, and other system diseases; 4) patients with oral non-steroidal anti-inflammatory drugs or glucocorticoids were excluded; 5) recent history of trauma or head surgery; 6) years of education ≤3.

We collected clinical data regarding sex, age at onset, disease duration, personal history, chronic disease history, treatment regimen and motor complications through a standardized personal interview. We made a detailed record of chronic disease history, and the use of antiparkinson drug was recorded at the time of enrollment and L-DOPA equivalent daily doses (LEDD) were evaluated according to the guidelines (Tomlinson et al., 2010). The severity of motor symptoms was assessed using the MDS‐UPDRS Part II (motor aspects of experiences of daily living) and Part III (motor examinations) scores in the off-medication state; the H&Y stage was also used to evaluate the motor severity (Hoehn and Yahr, 1967; Movement Disorder Society Task Force on Rating Scales for Parkinson’s Disease, 2003). The severity of non-motor symptoms (NMSs) was assessed with the Non-Motor Symptom Scale (NMSS) (Chaudhuri et al., 2007). The Chinese version of the NMSS includes nine domains and 30 items, and it is a valuable measure to assess the frequency and severity of NMS in Chinese PD patients (Wang et al., 2009). The MDS‐UPDRS Part I score was also applied to evaluate the overall non-motor symptoms. Executive function was assessed with the frontal assessment battery (FAB) (Dubois et al., 2000), while the global cognitive function was evaluated with the Montreal Cognitive Assessment (MoCA) (Nasreddine et al., 2005). Depression was evaluated by the 24-item version of the Hamilton Depression Scale (HAMD) (Moberg et al., 2001), and we applied the Hamilton Anxiety Scale (HAMA) for anxiety assessment (Hamilton, 1959).

Peripheral blood samples were collected in a fasting state from each PD patient and HCs and sent to the laboratory center of our hospital for testing. Serum levels of TNF-α, IL-6, and IL-8, were examined. The levels of the inflammatory factors mentioned above were detected according to the kit instructions.

## Statistical analyses

The distribution of serum levels of TNF-α, IL-6, IL-8, were tested for normality using skewness and kurtosis, Shapiro–Wilk and Shapiro–Francia tests. The Student’s *t*-test was applied to analyze the difference in these inflammatory indicators between PD patients and controls. Chi square tests compared sex ratios between PD and control groups. All data were presented in the form of the mean ± standard deviation (SD). Bonferroni correction was applied to optimize for multiple tests. Spearman’s rho correlation was used to calculate the correlations between the detected PIA variables and the H&Y stage, while Pearson’s correlation was applied to evaluate the correlations with the age of onset, disease duration, FAB, MoCA, HAMD, HAMA, NMSS, UPDRS part I, UPDRS part II, and UPDRS part III. The regression analysis with stepwise selection was used to investigate the associations with clinical variables, with TNF-α, IL-6, or IL-8 level as an independent variable. The binary regression model used in this study used the presence of PD as the dependent variable, and the parameters, including age, gender, TNF-α, IL-6 and IL-8 were used as covariables. The area under the curve of IL-6 and TNF-α in the diagnosis of PD and HC; the corresponding sensitivity, specificity, and 95% confidence interval were calculated. SPSS 17.0 software was used for statistical analysis of data, and *p* < .05 was considered statistically significant.

## Results

The demographic features of the PD and HCs were listed in Table 1. In the 273 PD patients, the sex ratio (male/female) was 124/149, and the mean age at examination was 58.3 ± 11.5 years. There was no significant difference in gender and age at examination between the HCs and PD patients. The results of the detected inflammatory indicators in PD patients and HCs were summarized in Table 1.

**TABLE 1:** Comparison of peripheral immune variables in PD patients and matched controls.

	**PD patients n = 273**	**HCs n = 91**	*p-value*
**Age at examine**	58.3 ± 11.5	58.7 ± 4.9	0.8137
**Male/female**	124/149	37/54	0.4658
**IL-6**	2.297 ± 1.494	1.915 ± 1.293	**0.024**
**IL-8**	11.49 ± 13.29	9.43 ± 5.00	1.503
**TNF-α**	6.268 ± 2.702	4.949 ± 2.163	**< 0.001**

HCs: Health controls; Bold values mean that the difference is statistically significant.

Analysis of inflammatory indicators showed that PD patients had significantly higher levels of IL-6 and TNF-α than HCs. However, IL-8 levels showed no significant difference in PD patients and HCs (Table 1). These results were obtained with the use of multiple corrections. We further studied the correlation between the levels of inflammation indicators and clinical variables by using Pearson’s or Spearman’s correlation analysis. The results showed that in PD patients, IL-6 level was found to be positively correlated with HAMD, and NMSS, UPDRS part I, part II, and part III, but IL-6 level in PD patients was inversely correlated with FAB and MoCA scores (Table 2). PD patients who had a higher serum IL-6 level were more likely to experience more severe motor and non-motor symptoms, depression, and cognitive impairment. Furthermore, serum TNF-α level positively correlated with age of onset and H&Y stage in PD patients (*p* = .006 and *p* = .037), and negatively correlated with FAB scores in PD patients (*p* = .010), but no associations were found with other clinical variables (Table 2). However, no associations were found between all the clinical variables and the serum IL-8 level (Table 2).

**TABLE 2:** Clinical characteristics of PD patients and the associations with inflammatory factor levels.

	PD patients	IL-6 *p-value*	IL-8 *p-value*	TNF-α *p-value*
age of onset (years)	55.2 ± 11.8	**0.026**	0.339	**0.006**
Disease duration (years)	3.1 ± 3.2	0.874	0.119	0.063
LEDD (mg/day)	162.5 ± 251.5	1.000	0.956	0.455
FAB	15.1 ± 2.6	**< 0.0001**	0.548	**0.010**
MoCA	23.4 ± 4.6	**0.024**	0.955	0.577
HAMD	8.6 ± 7.4	**< 0.0001**	0.588	0.749
HAMA	8.1 ± 7.0	0.276	0.288	0.714
NMSS total	25.6 ± 26.3	**< 0.0001**	0.990	0.154
UPDRS I	1.2 ± 1.7	**< 0.0001**	0.888	0.583
UPDRS II	8.0 ± 5.1	**0.010**	0.766	0.776
UPDRS III	27.9 ± 13.3	**0.003**	0.458	0.532
H &Y stage	2 (2, 2)	0.062	0.471	**0.037**

Bold values mean that the difference is statistically significant.

The potential clinical variables related to serum IL-6 level, including age of onset, FAB, MoCA, HAMD, NMSS-total UPDRS part I, part II, and part III were included in the stepwise linear regression model, and the results showed that serum IL-6 level was associated with MoCA score (*p* = .023), UPDRS I score (*p* = .023), but showed no associations with the remaining factors. These findings indicated that PD patients with more severe non-motor symptoms and heavier cognitive dysfunction had a higher IL-6 level. The binary logistic regression model indicated that IL-6 and TNF-α were associated with PD (*p* < .05). No significant correlations were found between the remaining clinical factors and PD.

**FIGURE 1:**
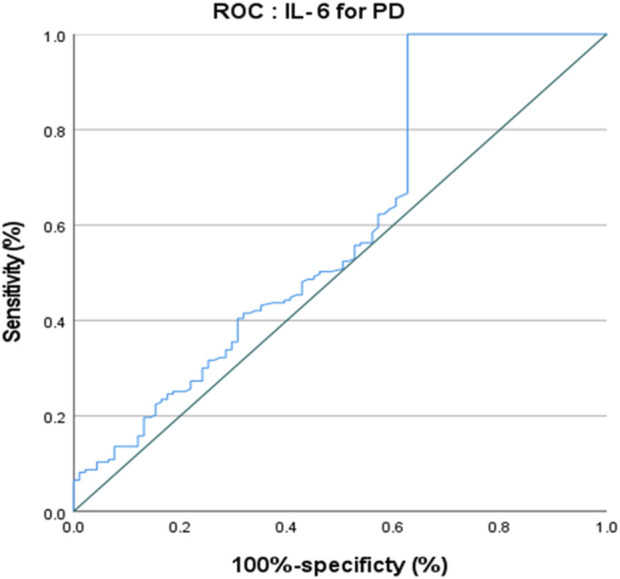
ROC curve of IL-6 for the diagnosis of PD.

**FIGURE 2:**
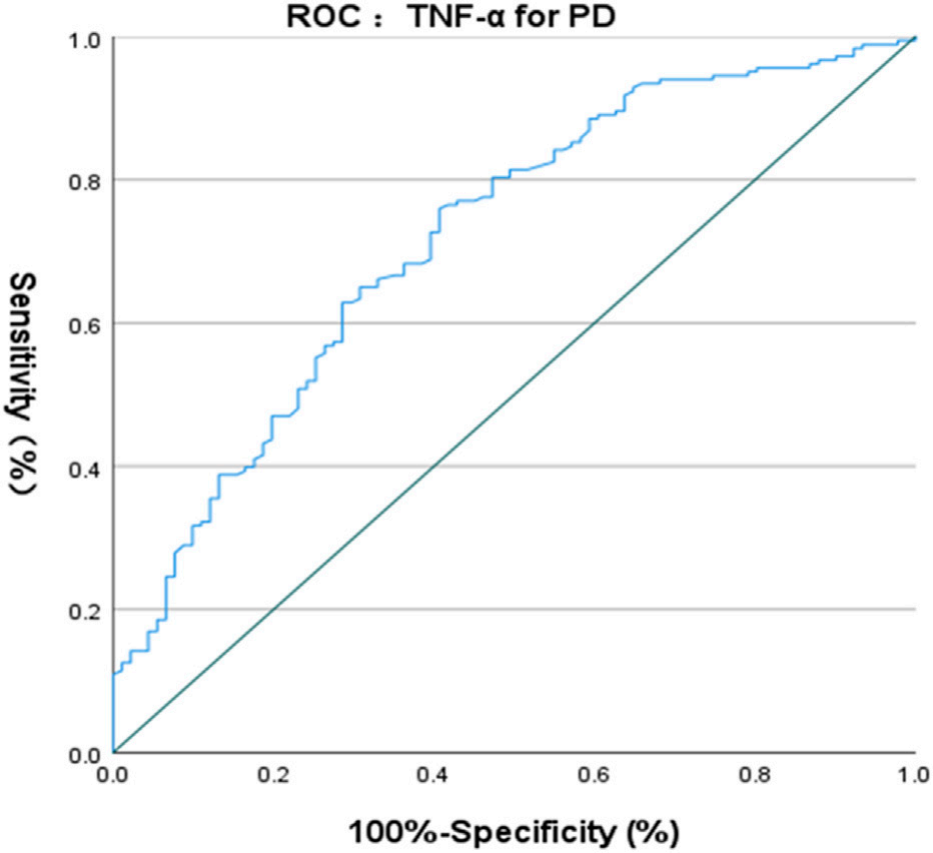
ROC curve of TNF- α for the diagnosis of PD.

**FIGURE 3:**
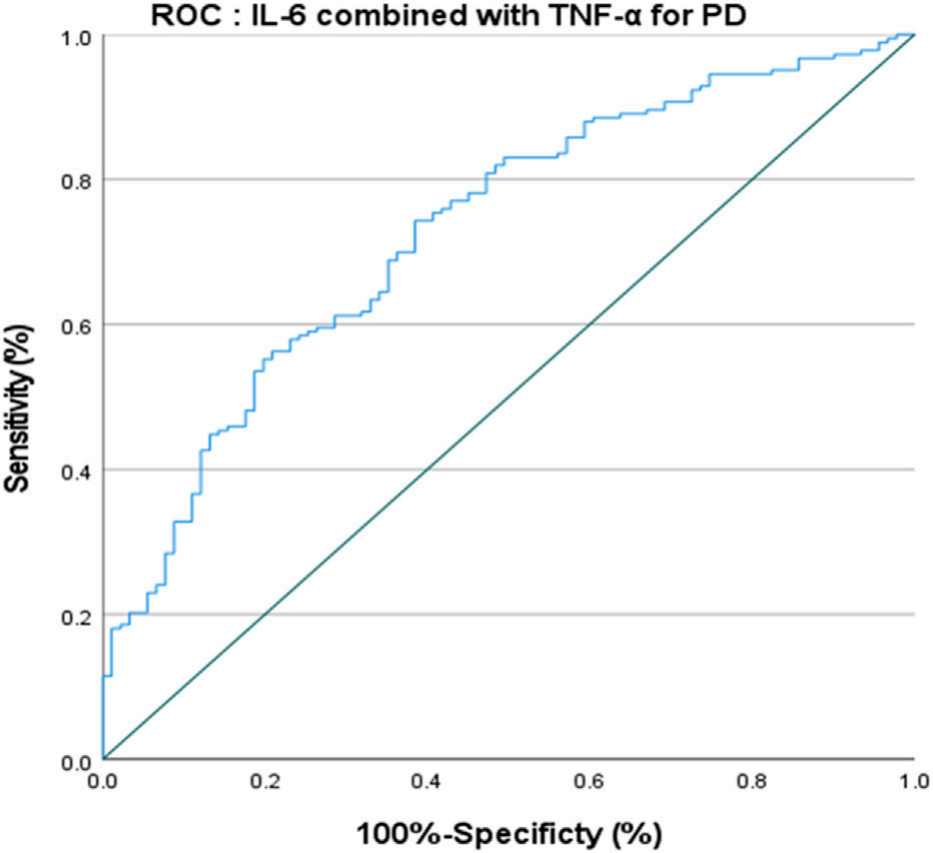
ROC curve of IL-6 combined with TNF- α for the diagnosis of PD.

The ROC curve of IL-6 for the diagnosis of PD showed that the area under the curve (AUC) was .599 (*p* < .05, 95% CI: .521–.676) indicating that single IL-6 has little value in the diagnosis of PD (Figure 1). The ROC curve of TNF-α for the diagnosis of PD showed AUC was .719 (*p* < .05, 95% CI: .655–.784), indicating that TNF-α has a good diagnostic value for PD, and the critical value of TNF-α for the diagnosis of PD was 5.380 pg/ml, with a diagnostic sensitivity of 76.0% and a specificity of 59.3% (Figure 2). The ROC curve for IL-6 in combination with TNF-α in the diagnosis of PD showed AUC .730 (*p* < .05, 95% CI: .668–.792). The sensitivity was 74.30% and the specificity was 61.50%, indicating that the value of TNF-α combined with IL-6 in the diagnosis of PD was basically the same as that of TNF-α alone (Figure 3).

## Discussion

Numerous studies have shown the involvement of neuroinflammation in the pathogenesis of PD, and increased proinflammatory cytokines were found in the CSF (Parnetti et al., 2013; Nagatsu et al., 2000) or blood (Williams-Gray et al., 2016) of PD patients. Activated microglia can secrete several proinflammatory cytokines, including TNF-α, IL-1β, and IL-6 (Hofmann et al., 2009; Scalzo et al., 2009; Qin et al., 2016; Williams-Gray et al., 2016; De Lella Ezcurra et al., 2010). The leakage of inflammatory factors from degenerated brain regions can also be detected in the peripheral blood. The changes in inflammatory biomarkers in the blood of PD patients also indicate the involvement of peripheral immunity in PD pathogenesis. Peripheral inflammatory molecules, such as IL-1β, IL-2, IL-6, IL-10, TNF-α, and high-sensitivity C-reactive protein (hs-CRP), could be used as potential biomarkers to reflect the neuroinflammatory pathogenesis of PD. These potential peripheral biomarkers would promote therapeutic intervention in the early stage of PD.

IL-6 is a multifunctional cytokine secreted mainly by neurons and glial cells, which plays a crucial role in the development and differentiation of neurons (Borsche et al., 2020). A study has found that circulating mitochondrial DNA (mtDNA) and interleukin-6 (IL-6) levels in PD patients with Parkin or PINK1 gene mutations are significantly increased, and IL-6 is considered to play a very critical role in the development of PD (Sliter et al., 2018). The basic mechanism of PD is that mutations in the PINK1/Parkin signaling pathway cannot allow damaged mitochondria to undergo mitophagy and be cleared, leading to the death of nerve cells in the substantia nigra. High expression of IL-6 was found to be detectable in both *pink1*−/− gene-deficient and *Prkn*−/− gene-deficient mice (Erta et al., 2012). These mechanistic studies provide a reference basis for IL-6 as a biomarker of PD. IL-6 level was also found to be elevated in the striatum, CSF, and serum of PD patients in many clinical studies (Dursun et al., 2015; Lindqvist et al., 2012; Müller et al., 1998; Mogi et al., 1994; Blum-Degen et al., 1995; Tang et al., 2014; Gruden et al., 2012; Dobbs et al., 1999; Brodacki et al., 2008). However, other studies did not report an increase in IL-6 level in the serum of PD patients (Stypuła et al., 1996; Koziorowski et al., 2012; Green et al., 2019; Dursun et al., 2015; Williams-Gray et al., 2016). In the present study, we also found that the serum IL-6 level was increased in PD patients, compared to age- and gender-matched normal controls. This finding was consistent with a 2016 meta-analysis, which included 13 studies (898 PD patients), and found a higher peripheral IL-6 level in PD patients (Qin et al., 2016). The mixed cohort of PD patients may cause inconsistency; the included patients may range from *de novo* patients to those with severe motor symptoms, and the heterogeneity would be large when all samples are grouped as PD patients. Therefore, more well-designed studies are required before a positive association can be conclusively established.

Serum IL-6 level was reported to be correlated with some clinical parameters, but the findings were inconsistent. For motor symptoms, previous studies found that serum IL-6 level was inversely correlated with functional mobility and gait speed in PD patients (Selikhova et al., 2002; Scalzo et al., 2009). However, another study has found that the serum IL-6 level was positively correlated with age, UPDRS part III score and H&Y stage (Gruden et al., 2012). Furthermore, Scalzo et al. (2009) reported that serum IL-6 level was not correlated with the UPDRS part III score and H&Y stage, which could reflect motor severity in PD (Tang et al., 2014). In the present study, we found that in Pearson’s correlation analyses, IL-6 level positively correlated with UPDRS part III score, but in the binary logistic regression model, this positive association was no longer in existence. Regarding the non-motor symptoms of PD, a previous study evaluated the non-motor symptoms using UPDRS part I and found that plasma IL-6 level was correlated with the severity of depression (Selikhova et al., 2002). PD patients with higher serum IL-6 levels at baseline had worse depression scores 2 years later (Veselý et al., 2018). And serum IL-6 level was also found to be inversely correlated with Mini-Mental Status Examination (MMSE) scores in PD patients (Selikhova et al., 2002; Scalzo et al., 2009). Similarly, the serum IL-6 level of PD patients was also negatively correlated with cognitive function scale score in PD patients (Veselý et al., 2019). Interestingly, a recent study investigated IL-6 level in PD patients with different gender and found that IL-6 positively correlated with a motor score in male patients, while higher IL-6 level was associated with worse cognitive performance in female patients (Green et al., 2019). IL-6 level was also found to be a significant predictor of fatigue scores in PD patients (Pereira et al., 2016; Green et al., 2019), and it was negatively correlated with the scores of the activity daily living scale in PD patients (Hofmann et al., 2009). Elevated serum IL-6 level was also associated with mortality in PD patients, and increased IL-6 levels may be a major factor in PD mortality (Dufek et al., 2015). In the present study, we found that serum IL-6 level positively correlated with age of onset, HAMD, and NMSS, UPDRS part I, UPDRS part II and UPDRS part Ⅲ, but negatively correlated with FAB and MoCA scores in PD patients. Furthermore, results of the stepwise linear regression proved again that serum IL-6 level was associated with MoCA and UPDRS I scores in PD patients, and this finding suggests a link between non-motor symptoms and periphery inflammatory processes. One study found that the neuroprotective effect of microglia regeneration and the cognitive dysfunction it caused were mediated by the IL-6 transduction signal (Willis et al., 2020). Serum IL-6 serum level may have biomarker potential to recognize non-motor symptoms and cognitive dysfunction in PD patients. However, the ROC curve of IL-6 for the diagnosis of PD showed that the AUC was .599, and the value of IL-6 in the diagnosis of PD was not significant. Therefore, IL-6 was more significant for the evaluation of non-motor symptoms of patients after the diagnosis of PD.

TNF-α is a proinflammatory cytokine that plays a key role in host defense (Tracey et al., 1994), and it is upregulated in the substantia nigra (SN) and CSF of PD patients (Boka et al., 1994; Mogi et al., 1994). TNF-αis an effective mediator of microglia function, and its neurotoxicity could lead to mitochondrial dysfunction and thus promote the progression of PD. TNFα could drive the death of oligodendrocytes and neuronal cells by activating pro-apoptotic pathways and increasing the misfolding and aggregation ofα-synuclein. TNF-α also plays an important role in angiotensin-induced dopaminergic cell death (Leal et al., 2013; Borrajo et al., 2014). Furthermore, treatments decreasing IL-1β and TNFα cytokine levels significantly improved motor function in a mouse PD model (Ndayisaba et al., 2019). The involvement of TNF-α in the pathogenesis of PD provides clues for its use as a biomarker for the diagnosis of PD. Serum TNF-α level was found to be elevated in PD patients (Müller et al., 1998; Gruden et al., 2012; Koziorowski et al., 2012; Lindqvist et al., 2012; Williams-Gray et al., 2016; Dobbs et al., 1999) and a meta-analysis in 2016 included nine studies (809 PD patients) also confirmed increased serum TNF-α level in PD (Qin et al., 2016). All these studies have illustrated the important role played by TNF-α in the pathogenesis of PD and provided clues for its use as a biomarker for the diagnosis of PD. Similarly, in the present study, we also found that serum TNF-α level was significantly higher in PD patients than in HCs, and we also found that TNF-α had a fairly good diagnostic value. A previous study found that TNF-α plasma level positively correlated with age in PD patients (Green et al., 2019). We also showed a positive association between age of onset and serum TNF-α level. Previous studies have shown that serum TNF-α level was negatively correlated with cognitive function scale score (Zhou et al., 2021), and positively correlated with H&Y stage (Kouchaki et al., 2018). These findings were consistent with the results of the present study. Previous studies found that plasma TNF-α level was positively correlated with cognitive impairment, depression, and disability in PD patients (Menza et al., 2010; Lindqvist et al., 2012), and it was also positively correlated with anxiety (Wang et al., 2016), age, and disease duration (Kouchaki et al., 2018; Li et al., 2018). However, the present study did not find that other motor and non-motor variables were associated with serum TNF-α levels. A previous study found that the levels of TNF-αin PD patients were significantly higher than those in the control group, but this longitudinal study also showed that IL-6 was related to the higher UPDRS-III motor score, and TNF-αwas related to the faster rate of motor function decline, rather than the baseline score of UPDRS-III. Therefore, longitudinal studies are required to verify the correlation of TNF-αwith clinical symptoms of patients with PD (Williams-Gray et al., 2016). Of course, there is also a possibility that TNF-αmay be non-linearly related to the clinical symptoms of PD, or may be related to differences in inclusion/exclusion criteria and evaluation tools. Despite the negative results of stepwise linear regression for the association between TNF-α and clinical symptoms of PD, the diagnostic value of TNF-α for PD should not be ignored. The ROC curve of TNF-α for the diagnosis of PD showed that the AUC was .719, indicating that TNF-α had a decent diagnostic value for PD, with a sensitivity of 76.0% and a specificity of 59.3%.

In the present study, serum IL-8 level failed to reach statistical significance; the relatively small number of HCs may partially explain this. Numerous previous studies investigated the serum IL-8 level in PD. However, the results are inconsistent. Williams-Gray et al. (2016) found no statistically significant difference in serum IL-8 level between PD patients and HCs, but Gupta et al. (2016) showed that the serum IL-8 level in PD patients was lower than that in HCs and the difference was statistically significant. Serum IL-8 level was also positively related to the disease duration, depression, and UPDRS III of PD patients (Gupta et al., 2016; Ahmadi Rastegar et al., 2015). In our study, serum IL-8 level did not correlate with motor or non-motor function in PD, which may be related to different serum IL-8 level detection methods, ethnic differences of patients, and inclusion criteria.

There are some limitations that cannot be ignored. First, the main limitation was the sample size in both the PD group and the control group. Second, it is impossible to establish a causal relationship between these inflammatory indicators and other clinical variables using a cross-sectional study design; the further prospective study will help elucidate this issue. Third, the differences in detection assay types, the present study used ELISA assay, but SIMOA is more highly sensitive than commercially available ELISA kits. However, the strengths of this study include the strict exclusion criteria and various assessment tools for non-motor symptoms.

## Conclusion

Our results suggest increased serum levels of IL-6 and TNF-α in PD, and we further found the correlation of IL-6 levels with non-motor symptoms and cognitive dysfunction. This finding suggests that IL-6 may play a role in the pathophysiology of non-motor symptoms in PD, and reflects a link between non-motor symptoms and periphery inflammatory processes. At the same time, we also propose that TNF-α has a good diagnostic value for PD despite its irrelevance to clinical symptoms.

## Data Availability

The original contributions presented in the study are included in the article/Supplementary Material, further inquiries can be directed to the corresponding authors.
